# Ontogenesis of the HPI axis and molecular regulation of the cortisol stress response during early development in *Dicentrarchus labrax*.

**DOI:** 10.1038/srep05525

**Published:** 2014-07-02

**Authors:** A. Tsalafouta, N. Papandroulakis, M. Gorissen, P. Katharios, G. Flik, M. Pavlidis

**Affiliations:** 1 University of Crete, Department of Biology, P.O. Box 2208, GR-714 09, Heraklion, Crete, Greece; 2 Hellenic Center for Marine Research, Institute of Aquaculture, P.O. Box 2214, Heraklion, Crete, Greece; 3 Department of Animal Physiology, Institute for Water and Wetland Research, Radboud University Nijmegen, Heyendaalseweg 135, 6525AJ, The Netherlands

## Abstract

The cortisol stress response and the molecular programming of the corticoid axis were characterized for the first time during early ontogeny in a Mediterranean marine teleost, the European sea bass (*Dicentrarchus labrax*). Sea bass embryos, pre-larvae and larvae at specific points of development were exposed to acute stressors and the temporal patterns of cortisol whole body concentrations and the expression of genes involved in corticosteroid biosynthesis, degradation and signaling were determined. Expression of genes (*gr1*, *gr2*, *mr*, *crf*) involved into the corticoid response regulation combined with histological data indicated that, although a cortisol stress response is evident for the first time around first feeding, a pattern becomes established in larvae at flexion until the formation of all fins. Moreover, mRNA transcript levels of *11β-hydroxylase* and *11β-hsd2* showed a strong correlation with the whole body cortisol concentrations. Concluding, our data reveal the presence of an adaptive mechanism in European sea bass at early ontogeny enabling to cope with external stressful stimuli and provide a better insight into the onset and regulation of the stress response in this species.

The teleostean hypothalamic–pituitary–interrenal (HPI) axis is a system comparable with the mammalian stress axis (hypothalamus-pituitary-adrenal; HPA), as a result of convergent evolution^1^,^2^ and it is of utmost importance in stress regulation as well as for the adaptation and/or acclimation of fish to their dynamic environment. Stress response includes the primary response, resulting in the rapid increase of circulating catecholamines and cortisol, the secondary leading to changes in several haematological and biochemical parameters, and the tertiary response that involves alterations at the whole animal and population level^1^,^3^,^4^. The fish's response to stressors may be of either an adaptive nature, allowing for homeostatic recovery, or a maladaptive nature having adverse effects on survival, growth, immune response, reproductive capabilities, behavior and general fitness^1^,^3^,^5^,^6^.

Exposure to stress can have a profound impact on the physiology and health of an organism later in life^7^,^8^. Studies carried out in mammals have shown that glucocorticoids play a key role in the programming of brain structures that can alter the responsiveness to stress^9^,^10^,^11^. In fact, it has been shown that exposure to stressors during development results in permanent changes in stress coping phenotypes in mammals^9^,^12^, birds^13^, amphibians^14^, and fish^15^.

In teleostean fish, cortisol is the principal corticosteroid and plays an important role in a number of physiological processes including growth, immunoregulation, maintenance of energy balance, and reproduction^2^,^16^,^17^,^18^. During HPI axis activation corticotropin-releasing factor (CRF), produced in the hypothalamic preoptic area (POA), stimulates the pituitary gland corticotropes to secrete adrenocorticotropic hormone (ACTH), which regulates cortisol synthesis and secretion. In teleosts, cortisol plays also a vital role in the maintenance of hydromineral balance, as fish cannot synthesize aldosterone, and cortisol carries out this mineralocorticoid function^1^,^19^. Cortisol enters by passive diffusion into the cells where its action is mediated by the Glucocorticoid receptor(s) (GR) and the mineralocorticoid receptor (MR)^20^, a class of ligand-activated transcription factors. During larval development, marine teleosts undergo dramatic changes in morphology, growth and metabolism in order to accomplish their metamorphosis into juvenile fish. Throughout this period, cortisol regulates osmoregulatory function^21^,^22^ and is implicated in the metamorphosis from larvae to juveniles^23^,^24^.

Studies conducted in European sea bass, *Dicentrarchus labrax*, and other species showed the presence of maternal cortisol in embryos and that *de novo* cortisol synthesis starts shortly after hatching but a significant elevation in whole body cortisol in response to a stressor becomes obvious days to weeks later, depending on the species^25^,^26^,^27^,^28^,^29^,^30^.

However, our knowledge on the development of the hypothalamic–pituitary– interrenal (HPI) axis of European sea bass and its response to stressors during early ontogeny or the molecular mechanisms involved is scarce. To this end, we examined the temporal patterns of cortisol and genes related to the corticosteroid signaling (*gr1*, *gr2*, *mr*, *crf*), corticosteroid synthesis (*11β-hydroxylase*) and cortisol metabolism or inactivation (*11β-hsd2*) at various stages during early ontogeny in order to assess the ontogenesis of the corticosteroid-signaling pathway. Moreover, we subjected European sea bass embryos, pre-larvae and larvae to an acute stressor in order to determine any differences in the timing or magnitude of the activation of the corticosteroid stress axis and the molecular response at each developmental point/stage.

## Results

### Ontogeny of the HPI axis in European sea bass

**Figure 1:**
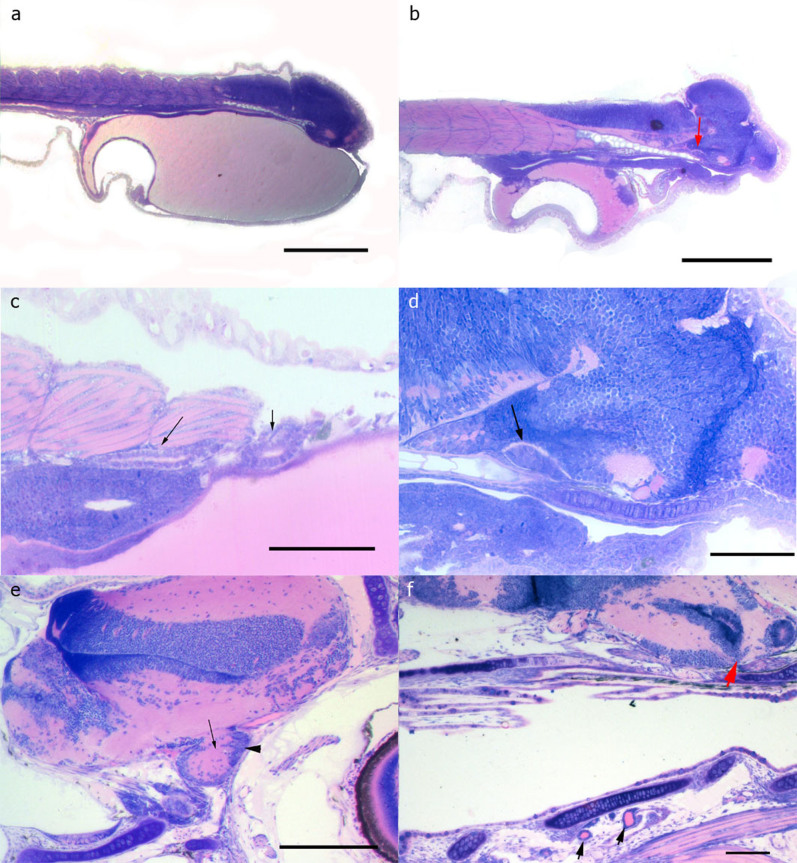
Histological analysis (a) Sea bass larvae at day 1 post hatching (dph). The brain covers the majority of the head area. Bar: 400 μm. (b) A sea bass larva at 5 dph with the pituitary morphologically differentiated (red arrow). Bar: 400 μm, (c) Kidney of a sea bass at 3 dph showing the distinct morphology of the kidney tubules (arrows). Bar: 100 μm, (d) higher magnification of picture (b) with the hypothalamus and the pituitary (arrow). Bar: 100 μm. (e) Brain of a 30 dph sea bass with fully differentiated pituitary. Arrow: Adenohypophysis, Arrowhead: Neurohypophysis. Bar: 100 μm. (f) Sea bass head at 30 dph. Red arrow points to the developed pituitary, black arrows point to thyroid follicles. Bar: 100 μm.

Histology data revealed that the brain of sea bass was evident at hatching (Figure 1a) while a morphological differentiation of the pituitary was possible at day 5 post hatching (dph) (Figures 1b, 1d). At this day the first thyroid follicles appear and they increase in number and size as fish grow (Figure 1f). Pituitary gland is clearly visible and differentiated at 30 dph when neurohypophysis and adenohypophysis are distinct (Figure 1e, 1f). Interrenal and chromaffin tissues are both located in head kidney adjacent to the cardinal vein. The kidney was present at 3 dph and it was characterized by the presence of kidney tubules with a distinct morphology (Figure 1c). However, morphological differentiation of the interrenal and chromaffin tissues was difficult at early stages of development using routine histology, and only at 28 dph, *i.e.* around flexion the respective endocrine cells were simultaneously evident.

### Temporal patterns of cortisol content and gene expression at early ontogeny

**Figure 2:**
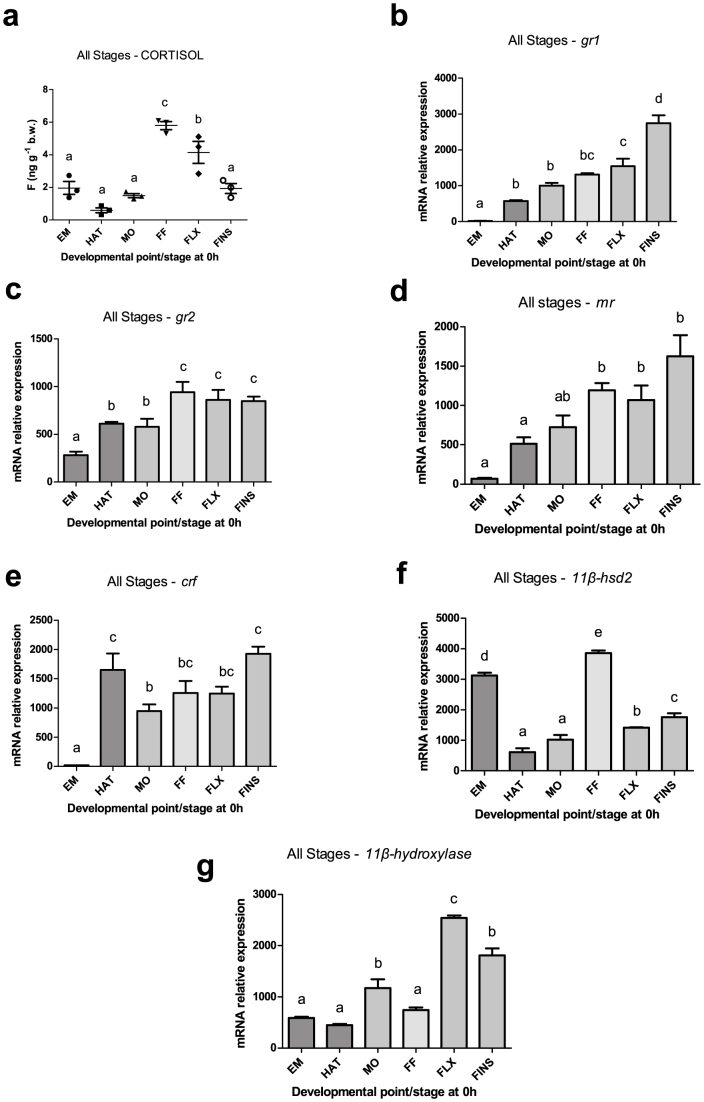
Temporal patterns of cortisol content and gene expression at early ontogeny. Changes in resting (0 h) whole body cortisol levels and mRNA transcript levels of *gr1*, *gr2*, *mr*, *crf*, *11β-hsd2* and *11β-hydroxylase* during the different developmental points/stages (embryos-EM, hatch-HAT, mouth opening-MO, first feeding-FF, flexion-FLX, formation of all fins-FINS). Values are means ± standard error (n = 3). Means with different letters differ significantly from one another (*P* < 0.05).

European sea bass embryos had low basal cortisol content (2 ± 0.7 ng g^−1^) that had declined at hatching (0.6 ± 0.3 ng g^−1^) and subsequently slightly increased at mouth opening (1.5 ± 0.2 ng g^−1^), but the differences were not statistically significant. The first peak (*P* < 0.001) was observed at first feeding (6.8 ± 1.3 ng g^−1^), after which whole-body cortisol mean concentrations dropped gradually from flexion (4.1 ± 1.2 ng g^−1^; *P* < 0.05) onwards to the formation of all fins (1.9 ± 0.5 ng g^−1^) (Figure 2a). All genes assessed in the current study were expressed in all developmental stages examined. Transcripts of *gr1* (Figure 2b) showed a gradual increase throughout early development, with lowest mRNA abundance recorded in embryos and highest at the formation of all fins. Expression of *gr2* (Figure 2c) was higher in embryos than *gr1* and showed a statistically significant increase (*P* < 0.05) at hatching and mouth opening, followed by a second increase at first feeding until the formation of all fins (*P* < 0.001). The mRNA abundance of *mr* (Figure 2d) showed a similar pattern to *gr1* with minimum levels in embryos, a slight increase at hatching and mouth opening and a statistically significant increase (*P* < 0.001) at first feeding until the formation of all fins. Expression of *crf* (Figure 2e) was detected in low levels in embryos, and then a bimodal pattern was observed with a statistically significant higher level of transcripts at hatching (*P* < 0.001) and the formation of all fins than at mouth opening (*P* < 0.05). *11β-hydroxylase* mRNA (Figure 2f) showed low levels in embryos, hatching and first feeding, a statistically significant increase (*P* < 0.001) in mouth opening and the formation of all fins and a peak at flexion. Finally, transcript levels of *11β-hsd2* (Figure 2g) showed a bimodal pattern of changes with high level of transcript in embryos and at first feeding and lowest at the other examined points or stages of development.

### Ontogeny of the cortisol stress response and molecular onset of genes related to the HPI axis

**Figure 3:**
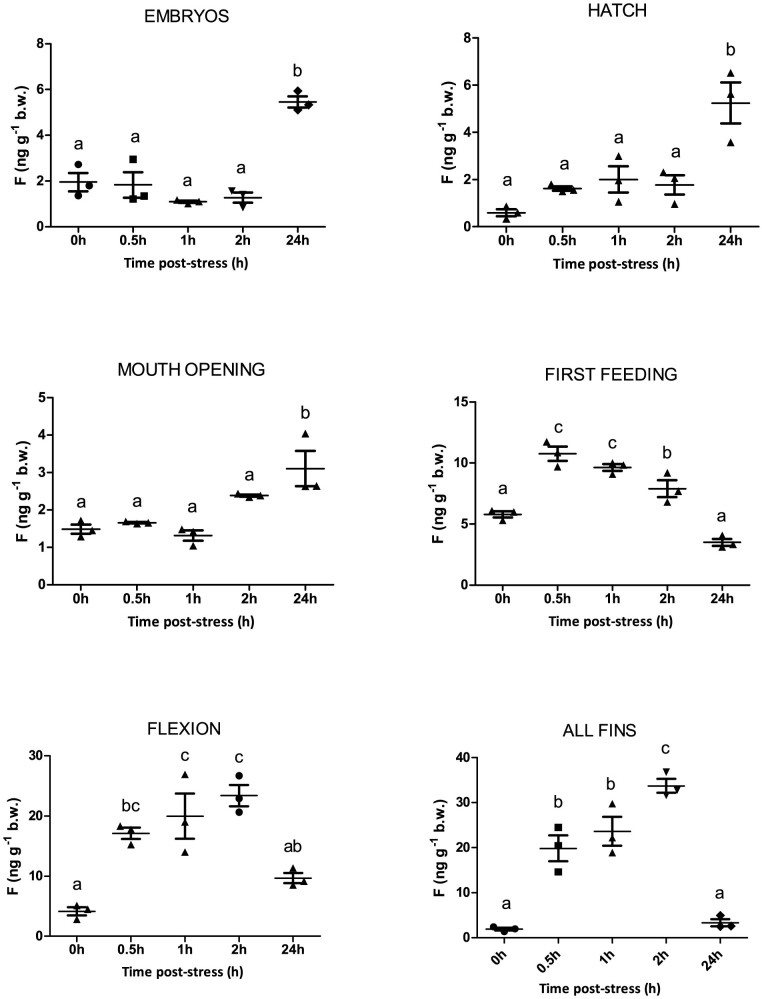
Ontogeny of the cortisol stress response. The cortisol response prior to (0 h) and after (0.5 h, 1 h, 2 h and 24 h) the application of the stressor during early ontogeny. Values are means ± standard error (n = 3). Means with different letters differ significantly from one another (*P* < 0.05).

**Figure 4:**
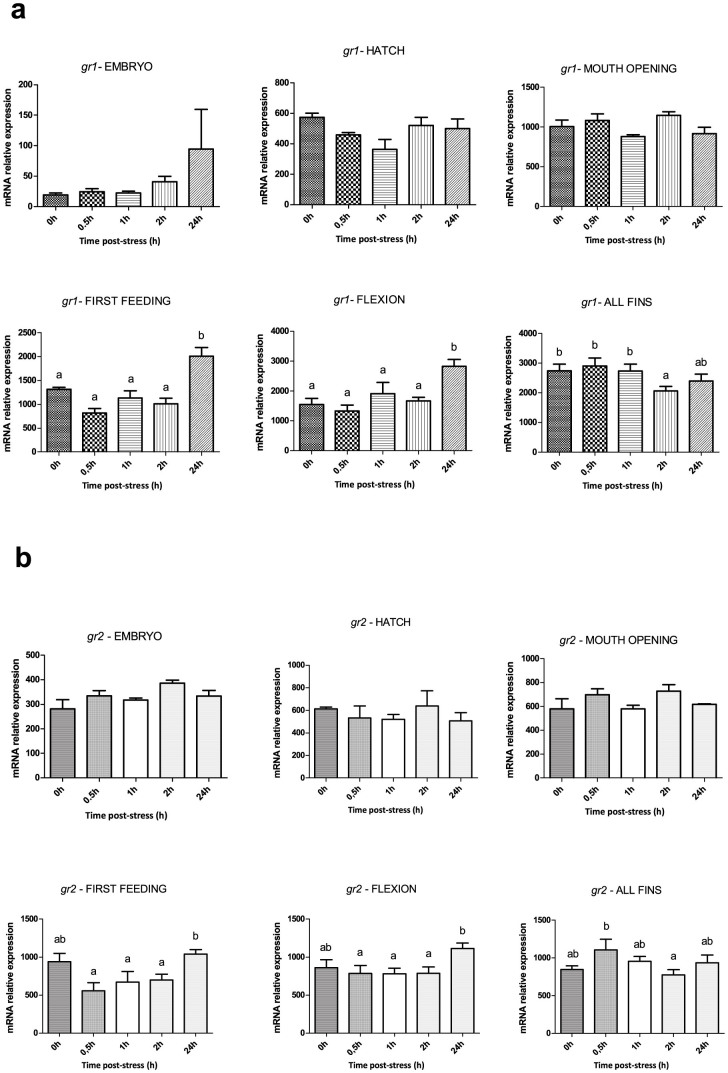
Expression of *gr1* and *gr2* after application of a stressor during early ontogeny. (a) Expression profile of *gr1* prior to (0 h) and after (0.5 h, 1 h, 2 h and 24h ) the application of the stressor at the different developmental points/stages. (b) Expression profile of *gr2* prior to (0 h) and after (0.5 h, 1 h, 2 h and 24 h) the application of the stressor at the different developmental points/stages. Values are means ± standard error (n = 3 pools of *ca.* 30 mg for embryos, hatched eggs and larvae samples, apart from juveniles where pools of 1–2 fish were used). Means with different letters differ significantly from one another (*P* < 0.05).

**Figure 5:**
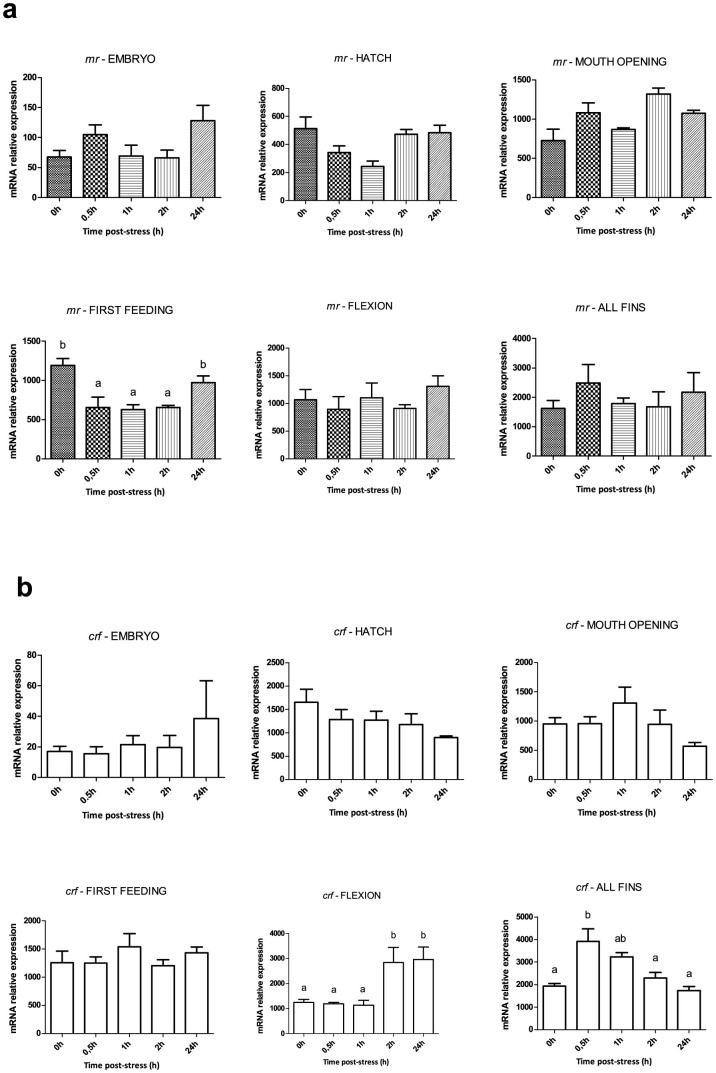
Expression of *mr* and *crf* after application of a stressor during early ontogeny. (a) Expression profile of *mr* prior to (0 h) and after (0.5 h, 1 h, 2 h and 24 h) the application of the stressor at the different developmental points/stages. (b) Expression profile of *crf* prior to (0 h) and after (0.5 h, 1 h, 2 h and 24 h) the application of the stressor at the different developmental points/stages. Values are means ± standard error (n = 3). Means with different letters differ significantly from one another (*P* < 0.05).

**Figure 6:**
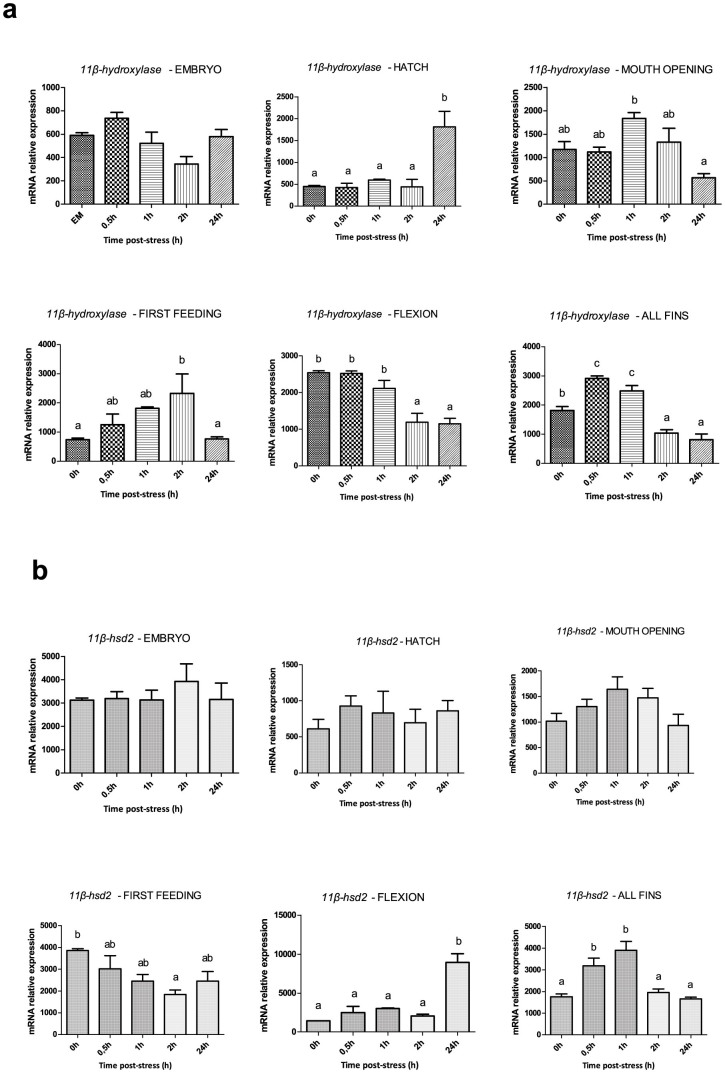
Expression of *11β-hydroxylase* and *11β-hsd2* after application of a stressor during early ontogeny. (a) Expression profile of *11β-hydroxylase* prior to (0 h) and after (0.5 h, 1 h, 2 h and 24 h) the application of the stressor at the different developmental points/stages. (b) Expression profile of *11β-hsd2* prior to (0 h) and after (0.5 h, 1 h, 2 h and 24 h) the application of the stressor at the different developmental points/stages. Values are means ± standard error (n = 3). Means with different letters differ significantly from one another (*P* < 0.05).

Figures 3 shows the cortisol response and Figures 4–6 the expression profile of the different stress-related genes prior to (0 h) and after (0.5 h, 1 h, 2 h and 24 h) the application of the stressor during early ontogeny (embryos, hatch, mouth opening, first feeding, flexion and formation of all fins). There was no statistically significant effect of stress on cortisol levels of embryos at 0.5 h, 1 h and 2 h post-stress, while there was a significant (*P* < 0.001) increase at 24 h after the application of the stressor, *i.e.* approximately 48 h following fertilization. An identical pattern of changes was observed after stress at hatching and mouth opening. A statistically significant (*P* < 0.05) effect of the stressor on whole-body cortisol concentrations soon after the application of the acute stressors was observed for the first time at first feeding, where whole body cortisol increased from the 0 h basal values (6.8 ± 1.3 ng g^−1^) to a maximum at 0.5 h (10.8 ± 1.0 ng g^−1^) and 1 h (9.6 ± 0.5 ng g^−1^) to return to basal values at 24 h (3.5 ± 0.5 ng g^−1^). At flexion, a prolonged peak was observed with basal cortisol values at 0 h (4.1 ± 1.2 ng g^−1^) and peak values (*P* < 0.05) at 0.5 h (17.1 ± 1.7 ng g^−1^), 1 h (19.9 ± 6.5 ng g^−1^) and 2 h (23.4 ± 3.1 ng g^−1^) post stress. In addition, the magnitude of the stress response was higher (*P* < 0.001) than the respective at first feeding. Finally, at the formation of all fins, cortisol content at 0 h (1.9 ± 0.5 ng g^−1^) sharply increased at 0.5 h (17.1 ± 1.6 ng g^−1^) to reach a maximum at 2 h (33.7 ± 2.7 ng g^−1^) after stress and return to basal values at 24 h (3.3 ± 1.4 ng g^−1^). The magnitude of the stress response was statistically significant higher (*P* < 0.001) than the respective in first feeding and flexion. Transcripts of *gr1* (Figure 4a) showed no statistically significant changes following exposure to the stressors at hatching and mouth opening. However, in first feeding and flexion there was a 1.9- and 1.8-fold upregulation (*P* < 0.001) at 24 h, respectively, while at the formation of all fins a significant 1.4-fold downregulation (*P* < 0.05) compared to controls was observed at 2 h post-stress. *A* similar pattern of expression was observed in *gr2* transcripts (Figure 4b), with stable mRNA abundance during hatching and mouth opening and an upregulation (*P* < 0.05) at 24 h at first feeding and flexion. In the formation of all fins, *gr2* reaches a maximum at 0.5 h after stress and then mRNA levels fall down to a minimum at 2 h. Transcript levels of *mr* (Figure 5a) showed no statistically significant changes between the different time points, apart at first feeding were a decrease is observed from 0.5 h till 2 h after stress. Exposure to stressors did not affect *crf* abundance around hatching, mouth opening and first feeding, however, in flexion there was an upregulation (*P* < 0.001) at 2 h and 24 h post-stress. In all fins, *crf* transcripts peaked, as whole-body cortisol concentrations, at 0.5 h post stress followed by a gradual decrease to the basal levels of control at 24 h (Figure 5b).

*11β-hydroxylase* expression pattern showed statistically significant temporal differences at all points/stages of development. In particular, at hatching there was a significant increase (*P* < 0.001) at 24 h (*i.e.* hatching 100% completed) post-stress, while at mouth opening and first feeding there was a peak at 1 h and 2 h post-stress respectively. In flexion, *11β-hydroxylase* abundance remained at high levels from 0 h till 1 h post stress after which there was a statistically significant decrease (*P* < 0.05) at 2 h. In all fins, the pattern of changes resembled that of cortisol, with low transcripts at 0 h, 1.6 to 1.4-fold upregulation at 0.5 and 1 h followed by a sharp downregulation at 2 h (Figure 6a). *11β-hsd2* levels did not show significant changes around hatching and mouth opening. However, in first feeding there was a gradual decrease in mRNA transcripts from high values at 0 h to a minimum at 2 h post-stress. In flexion, *11β-hsd2* abundance was constant except for a sharp increase at 24 h. However, if we exclude the values at 24 h, there was a statistically significant increase at 1 h post stress, which is no longer “masked” by the high values at 24 h. As in the case of *11β-hydroxylase*, at the formation of all fins the pattern of *11β-hsd2* changes are similar to that of cortisol, with peak values at 0.5 and 1 h post-stress followed by a drop to the basal values at 2 h and 24 h (Figure 6b).

## Discussion

The temporal changes of whole-body cortisol levels during the early developmental stages of teleosts show that the initial maternal deposit of cortisol is depleted during embryogenesis and reaches a minimum around the time of hatch and then the larva begins to synthesize cortisol *de novo*, a pattern which is observed in a similar way across a number of species. These results are in agreement with results obtained in this study as well as in studies in Japanese flounder^16^, tilapia^38^, rainbow trout^25^, Asian sea bass^39^, common carp^33^, gilthead sea bream^27^, Atlantic salmon^40^, zebrafish^28^, and in previous work in E. sea bass^30^.

*Gr1* is present in the embryos with very low transcript levels but soon after hatch its expression follows a continuous elevation during development. *Gr2* abundance in embryos is higher than *gr*1, and its expression pattern is characterized by an initial elevation in hatch and a second in first feeding followed by a relatively steady expression, thereafter. Previous immunohistochemical and *in situ* hybridization data also verify the presence of *gr2* mRNA transcripts and of the glucocorticoid receptor early in development with increasing expression towards the juvenile stage^41^. In addition we show, for the first time, the expression profile of *mr*, *crf*, *11β-hydroxylase* and *11β-hsd2* during the early stages of development. *Mr* is present in embryos in very low copies and its expression increases as development proceeds, following a pattern similar to that obtained for *mr* expression during embryogenesis in zebrafish^28^.

*Crf* expression is detected in embryos in very small quantities; however, there is a peak in mRNA transcripts at hatch that decline in mouth opening and then gradually increase from first feeding to the formation of all fins, indicating a maturation of the HPI axis. The expression of *11β-hydroxylase* is upregulated immediately before the rise in larval cortisol levels which occurs in first feeding, pointing to the activation of the steroidogenic pathway around that time. *11β*-*hsd2* mRNA transcripts follow a transient pattern as they appear at high levels in embryos, drop in hatch, reach a maximum in first feeding and then drop again at the following developmental stages. The high amounts in embryos may be associated with the maternal cortisol deposit that needs to be metabolized and the second peak which appears in first feeding coincides with the first peak of cortisol during early development of sea bass, and may reflect the immediate response of the corticoid system to the sudden accumulation of cortisol. Apart from embryos where the mRNA abundance of *11β-hsd2* and *11β-hydroxylase* is high, the expression patterns of these genes are quite similar to the respective patterns obtained from zebrafish^28^.

The acute stress challenge tests didn't result in a cortisol response in embryos, hatch and mouth opening stages, apart from a maximum at 24 h post stress. However, further research is needed to clarify whether this maximum is a result of a delayed stress response or reflects differences in the developmental point/stage. Histological data showed that the first appearance of a distinct hypothalamo-hypophysial-interrenal axis is observed at first feeding, where a peak in whole body cortisol levels was observed at 0.5 h post stress, followed by a protracted decrease until at 24 h when it reached resting levels. These results, in accordance with the molecular data, imply that as early as at first feeding sea bass individuals are capable of responding to external noxious stimuli. In addition the first peak observed in whole-body cortisol concentrations at first feeding reflects the essential role of cortisol to carbohydrate and protein metabolism towards transition to exogenous feeding. As development proceeds, the magnitude and duration of the response is higher and a pattern seems to be established from flexion until the formation all fins where cortisol values reach a maximum at 2 h. This is further supported by the histological data showing that while the kidney tubules with a distinct morphology were present at hatching, a clear morphological differentiation of the interrenal and chromaffin tissues was possible only at 28days post hatch (*i.e.* at flexion) using routine histology. Similar results were found in cichlid fishes where the head kidney from 12 to 30 days after fertilization is functionally mixed, with the nephron and developing hemopoietic and endocrine (chromaffin and interrenal tissue)^42^. These results indicate that even at the stage of first feeding fish are capable of a stress-induced stimulation of cortisol and that the HPI axis becomes gradually established until the development of all fins. This is in accordance with previous work conducted in European sea bass^30^, rainbow trout^25^, the yellow perch^26^ and the zebrafish^28^. However, this is the first time that the exact pattern of cortisol response following exposure to acute husbandry stressors is revealed at early developmental stages.

With the aim to shed light on the molecular mechanisms related to the onset of the cortisol stress response, we carried out qPCR experiments in order to measure the mRNA transcript levels of genes related to the HPI axis, *gr1* and *gr2*, *mr* and *crf*; and genes related to the biosynthesis and degradation of cortisol, *11β-hsd2* and *11β-hydroxylase*. During HPI axis activation, *gr1*, *gr2* and *mr* are the mediators of the transcriptional effects of circulating cortisol on target tissues and *crf* produced in the hypothalamic preoptic area (POA), stimulates the pituitary corticotropes to secrete adrenocorticotropic hormone (ACTH)^43^,^44^, which in turn stimulates synthesis and secretion of cortisol into the circulation^45^. *Gr1* expression levels after application of an acute stressor were not altered in any developmental stage apart from all fins, where a down-regulation was observed at 2 h post stress. The same is the case of the mRNA levels of *gr2*, as the only response to the stress was detected at the stage of all fins, where an increase was observed at 0.5 h post stress followed by a down-regulation at 2 h. The down-regulation of *gr1* and *gr2* is in accordance with data from other studies carried out in sea bass exposed to very high stocking densities^46^, in coho salmon^47^, in Atlantic salmon^6^, in common carp^48^, in the hippocampus of rats exposed to increasing corticosteroid levels^49^, in mouse pups with high corticosteroid levels due to 24-h maternal deprivation^50^. The statistically significant increase of the expression levels of both *gr1* and *gr2* observed at 24 h post stress in first feeding may be related, as with the case of cortisol, either to a delayed stress response or to the role of cortisol in metabolism and neural development for the passage of fish to exogenous feeding. The mRNA abundance of *mr* was not altered at any of the developmental stages examined apart from first feeding where the expression levels decreased from 0.5 h to 2 h post stress. However, as this is not repeated in the later stages and especially at all fins where the HPI axis is expected to be more mature, this alteration may not reflect a response to stress but rather a suppression related to the needs of the developmental stage. *Crf* expression pattern at all fins follows an increase at 1 h and a gradual decrease until 24 h post stress, which is in accordance with the pattern observed for cortisol at this stage, where the peak of cortisol levels is at 2 h post stress. In flexion, there is a statistically significant increase at 2 h that remains at high values still at 24 h, indicating that at this stage the *crf* system is being established, but the prolonged expression and the delayed response compared to the pattern of cortisol at the same stage, reveals that it is not mature yet.

During fish ontogenesis cortisol is a critical hormone when changes occur at the metabolic demands of the larvae^21^,^24^,^27^ and it is also implicated in neural development and in the induction metamorphosis^16^,^51^. The inability of the stress response system to respond to the stress-elevated cortisol levels via the *crf* and the *gr*s at these stages until only at all fins, might be of critical importance for the survival of the larvae and the normal progress of the development. There are very limited data available about the role of *gr* and *mr* in fish development. The major mineralocorticosteroid in mammals and non-mammalian vertebrates is aldosterone. However, in fish, deoxycorticosterone (DOC) is considered to be a *mr* ligand instead of aldosterone, as the latter is not detected in fish, but also cortisol is a high-affinity ligand for *mr*. A recent study carried out in zebrafish showed that both GR and MR are present during embryogenesis and suggested that *gr* plays a more important role after hatching in zebrafish, whereas *mr* is suggested to be important at the earlier stages of development, and that after hatching a ligand other than cortisol, perhaps DOC, may be responsible for *mr* signaling^28^. Studies in mice showed that mice lacking a functional *gr* survive until birth but die shortly thereafter due to impaired lung development^52^ and that there is no abnormal embryonic development detected in a mutant zebrafish line that does not develop corticotropic pituitary cells^53^. Other studies demonstrated that knocking down maternal *gr* leads to developmental defects in mesoderm formation in zebrafish^54^ and that *gr* signaling is essential for zebrafish muscle development^55^. In the present study, *mr* expression profiles during the stress response in first feeding seems to give *mr* a more important role at this stage than that of *gr*s, which at the later stages of development is inverted. This is in accordance with the results obtained from the other studies mentioned above.

We tested the hypothesis that the molecular events related to the appearance of the cortisol synthesis pathway are tightly linked to the enzymes which take part in cortisol biosynthesis and degradation. Therefore, we quantified the temporal expression of *11β-hydroxylase* that generates cortisol from 11-deoxycortisol^56^ and *11β*-*hsd2*, an enzyme that converts the biologically active cortisol to the inactive cortisone. After the acute stress application the transcript levels of *11β-hydroxylase* appear statistically significant altered at hatch and mouth opening but these changes may not reflect a stress response, but these changes could represent the role of this enzyme in gonad differentiation, as *11β-hydroxylase* appears to be a key transducer in the mechanism of sex determination in fish^57^. However, in first feeding appears a strong -relation between *11β-hydroxylase* transcripts and cortisol increase. This pattern continues also in the later stages of development, where mRNA expression of *11β-hydroxylase* is upregulated along with cortisol levels. This is in accordance with data obtained for rainbow trout, where mRNA abundance of *11β-hydroxylase* also increases in response to an acute stressor^58^. In first feeding, where the first response is observed, *11β*-*hsd2* mRNA transcripts are at high amounts in the larvae and gradually decline to a minimum at 2 h post stress; if compared with the cortisol pattern, it becomes clear that the mRNA levels of this enzyme are correlated with the amount of cortisol present. At the following developmental stages this becomes more obvious as the maximum mRNA levels of this gene are observed at 1 h post stress, just prior to the cortisol peaks (at 2 h), and at 2 h the transcript levels drop again to the resting values.

The cortisol stress response and the regulation of genes related to the corticoid axis in combination with histological analysis were studied for the first time during early ontogeny in European sea bass, *Dicentrarchus labrax*. Sea bass embryos, pre-larvae and larvae were exposed to acute stressors and the temporal patterns of cortisol whole body concentrations and the expression of genes involved in corticosteroid biosynthesis (*11β-hydroxylase*), degradation (*11β-hsd2*) and signaling (*gr1, gr2, mr* and *crf*) were determined. Histological data showed that although the kidney was present at hatching, morphological differentiation of the interrenal and chromaffin tissues was difficult at early stages of development using routine histology, and only around flexion the respective endocrine cells were simultaneously evident. Whole body cortisol concentrations showed a decline from embryos to hatching, remained at low levels at mouth opening and peaked at first feeding. In addition, around first feeding an effect of stress was evident for the first time on post-stress cortisol concentrations. As development proceeds, a pattern with a higher magnitude and longer duration of the cortisol stress response was established from flexion until the formation of all fins. Expression data of genes related to the regulation of the corticoid response (*gr1*, *gr2*, *mr* and *crf*) indicated that, although a cortisol stress response is evident at first feeding, the HPI axis seems to be fully mature only at the stage of all fins. Moreover, the mRNA transcript levels of *11β-hydroxylase* and *11β-hsd2* showed a strong correlation with the whole body cortisol concentrations. Overall, the data indicate that fish do respond to external noxious stimuli as early as at first feeding but the cortisol stress response becomes fully functional and mature until only at the stage of all fins. In addition, several changes occurred in early development (embryos, hatching and mouth opening) may resemble, apart from an adaptive to stress role of cortisol, its implication in other important aspects of development and metabolism. In conclusion, our data reveal the presence of an adaptive mechanism in European sea bass at early ontogeny enabling to cope with external stressful stimuli and provide a better insight into the onset and regulation of the stress response in this species.

## Methods

### Animals and husbandry conditions

Batches of fertilized European sea bass eggs were obtained from a private fish farm (DIAS S.A.) and transferred to the installations of the Institute of Aquaculture, Hellenic Center for Marine Research (Heraklion, Crete). Larval rearing was performed applying the pseudogreen-water technique^31^, in 500 L cylindro-conical tanks, with an initial density of 100 eggs L^−1^ in which both hatching and rearing took place. Tanks were coupled to a biological filter and were initially filled with filtered seawater from a deep well. Aeration was provided by means of a wooden diffuser located in the tank center at a rate of 150–200 ml min^−1^. Larvae were held during the whole experimental period under mean (±SD) water temperature of 18 (±1.6)°C, dissolved oxygen levels of 7.2 ± 0.8 mg l^−1^, salinity of 36 and pH of 7.9 ± 0.3. Food was delivered only when inflated swim bladder was observed in more than 80% of the population. Exogenous feeding was based on rotifers (*Brachionus* sp.) at 5 individuals ml^−1^ enriched with proteins and PUFA (INVE Aquaculture S.A., Belgium) until 10 days post hatching (dph) while phytoplankton (*Chlorella* sp.) was supplied until 10 dph. Enriched *Artemia* nauplii (Instar ΙΙ, EG, Artemia Systems S.A., Belgium) were delivered since 10 dph until 50 dph at 0.5 to 1.0 individual ml^−1^. From 30 dph, larvae were offered dry feed (PROTON 2–3, INVE Aquaculture S.A., Belgium) using automatic feeders. On day 50 dph, the type of the dry feed changed to PROTON 3–5 (INVE Aquaculture S.A., Belgium). During larval rearing and pre-weaning a sample of 10 larvae was taken daily to determine the morphological characteristics and record of total length while 2 times per week weight measurements were also performed with a sample of 10 individuals. The trial lasted until individuals completed the formation of their fins on 45 days post hatch (dph).

### Experimental design

**Figure 7:**
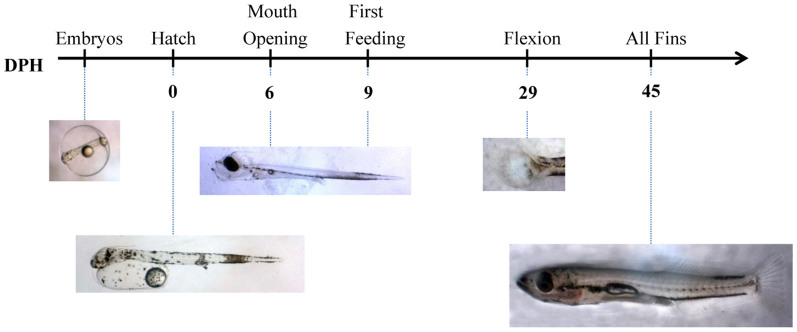
Sampling design. Ontogeny of the neuroendocrine stress response in European sea bass (DPH: Days Post Hatch). An acute stress test was applied in all developmental points/stages.

Samples were collected at six different embryonic and larvae phases (embryos, hatching, mouth opening, first feeding, flexion and formation of all fins; Figure 7/Table 1), prior to and after the application of an acute stress test. Different stressors were applied based on the tolerance of larvae at the particular developmental stages (Figure 7). In particular, embryos were exposed to transportation stress for 8 hours at a density of 50 g L^−1^ and then to netting and air exposure for 1 min until distribution to the incubation tanks. Pre-larvae (hatching, mouth opening, first feeding) were exposed to chasing with a net for 20 sec and high aeration (1,000–1,500 ml min^−1^ vs. 150–200 ml min^−1^) for 90 sec. Larvae (flexion and formation of all fins) were exposed to high aeration (as above), chasing with a net for 20 sec, confinement (collection in beakers), and air exposure for 5 sec before being transferred to baskets within a 500 L tank. Samples for molecular and endocrine (cortisol) analysis were collected with a net at 0, 0.5 h, 1 h, 2 h and 24 h post-stress, flash frozen in liquid N_2_ and stored at −80°C. All experiments were performed in accordance with relevant guidelines and regulations. The laboratories of the Hellenic Centre for Marine Research are certified and obtained the codes for breeding animals for scientific purposes (EL-91-BIO-04). Furthermore all procedures involving the handling and treatment of fish used during this study were approved by the HCMR Institutional Animal care and use committee following the Three Rs (3Rs, Replacement, Reduction, Refinement) guiding principles for more ethical use of animals in testing, in accordance to Greek (PD 56/2013) and EU (Directive 63/2010) legislation on the care and use of experimental animals.

### Histological analysis

Sea bass larvae were killed with an overdose of anesthetic (ethylene glycol monophenyl ether, Merck, 807291) and fixed in buffered formalin. Samples were dehydrated in a 70–95% ethanol series and embedded in glycol methacrylate resin (Technovit 7100, Heraeus Kulzer, Germany). Serial sections were obtained at a thickness of 3–5 μm on a microtome (Leica RM2245, Germany) using disposable blades. After drying, slides were stained with methylene blue/azure II/basic fuchsin^32^ and examined under a light microscope in order to record the first appearance of the tissues comprising the HPI axis and to describe the relevant organs/tissues.

### Whole body cortisol

Samples were homogenized according to Stouthart *et al.*^33^. Cortisol was measured in duplicate using a RIA in a 96-well plate according to Gorissen *et al.*^34^. All wells except the ‘non-specifics’ received 100 μl cortisol antibody (Cortisol Antibody[xm210] monoclonal and IgG purified (Abcam) and were incubated overnight at 4°C. Subsequently, the plates were washed three times with 200 μl/well wash buffer and 100 μl blocking buffer was added to each well in order to block the non-specific sites. Plates were covered and incubated for one hour at 37°C. After the incubation, 10 μl of standard (4 pg–2048 pg cortisol/10 μl) or 10 μl of undiluted homogenate was added to designated wells and 10 μl assay buffer was added to the non-specifics and B_0_. All wells received 90 μl (333 Bq) of 3H-hydrocortisone (PerkinElmer, USA) solution and plates were incubated at room temperature for 4 hours. The plates were then washed three times with wash buffer and after the final wash step, all wells received 200 μl of ‘Optiphase hisafe-3 scintillation liquid’ (PerkinElmer, USA). Beta-emission was quantified by a 3 min count per well using a Microbeta Plus (Wallac/PerkinElmer, USA).

### RNA purification and cDNA synthesis

Samples of embryos, pre-larvae and larvae were let to thaw on ice, disrupted and homogenized using the TissueRuptor (Qiagen, Hilden, Germany) for 20 s in 600 μl RLT plus buffer (RNeasy Plus Mini Kit Qiagen, Valencia, USA). Total RNA was isolated with the RNeasy Plus Mini Kit (Qiagen, Valencia, USA). RNA yield and purity was determined by measuring the absorbance at 260 and 280 nm using the Nanodrop® ND-1000 UV–Vis spectrophotometer (Peqlab, Erlangen, Germany), and its integrity was tested by electrophoresis in 1% agarose gels. Reverse transcription (RT) was carried out using 1 μg RNA with QuantiTect Reverse transcription kit (Qiagen).

### Primer design

Primers for Glucocorticoid Receptor 1 (*gr1*), Glucocorticoid Receptor 2 (*gr2*), Mineralcorticoid Receptor (*mr*), Corticotropin Releasing Factor (*crf*), eukaryotic Elongation Factor 1 (*eEF1a*), 40S Ribosomal protein S30 (*Fau*) and ribosomal 18S RNA (*18S*) were obtained by a previous work of our group^30^,^35^. Primer design for steroid *11β-*hydroxylase (*11β-hydroxylase*) was based on the available sequence with accession number AF449173.2^36^. Forward and reverse primers for 11-*β*-Hydroxysteroid Dehydrogenase type II (*11β-hsd2*) were designed based on the conserved regions as revealed by multiple sequence alignments of other teleost fish.

In the case of *11β-*hydroxylase, the forward primer (11β_Fwd) was a 21-mer with the sequence 5′ -GGAGGAGGATTGCTGAGAACG- 3′ and the reverse primer (11β_Rev) an 19-mer primer with the sequence 5′ -AGAGGACGACACGCTGAGA- 3′. For *11β-hsd2*, the sequence of the forward primer (hsd_Fwd) was 5′ -CACCCAGCCACAGCAGGT- 3′ and the reverse primer (hsd_Rev) had the sequnece 5′-ACCAAGCCCCACAGACC- 3′. The products of each primer pair were further checked with sequencing in order to confirm that they amplify the desired genes.

### Quantitative real-time PCR (qPCR)

The mRNA expression of genes encoding for *gr1*, *gr2*, *mr*, *crf*, *11β-hydroxylase* and *11β-hsd2* was determined with quantitative polymerase chain reaction (qPCR) assays using the *KAPA SYBR*® *FAST* qPCR Kit (Kapa Biosystems). Reactions were cycled and the resulting fluorescence was detected with MJ Mini Thermal Cycler (Bio-Rad) under the following cycling parameters: 95°C for 3 min (HotStarTaq DNA Polymerase activation step), 94°C for 15 s (denaturation step), 60°C for 30 s (annealing step), 72°C for 20 s (extension step), 40 cycles (step 2–step 4). Levels of *gr1*, *gr2*, *mr*, *crf*, *11β-hydroxylase* and *11β-hsd2* mRNA were normalized based on the reference genes *18S*, *eEF1a* and *Fau*. A relative standard curve was constructed for each gene, using 4 serial dilutions (1:5) of a pool of all cDNA samples. We also performed geNORM analysis^37^ in order to validate which are the most suitable reference genes to serve as an internal control and we concluded to *eEF1a* and *18S*.

### Statistical analysis

All statistical analyses were performed with SigmaPlot 11.0 (Jandel Scientific). All data are presented as means ± standard error of the mean (SEM). Data were initially screened for normality and homogeneity. Statistical comparisons of temporal patterns of cortisol and gene expression of unstressed specimens (0 h) between the different developmental stages were made using one-way ANOVA. Statistical comparisons of cortisol content and gene expression between the different time points following exposure to a stressor and the various developmental points/stages tested were made using two-way ANOVA. Holm-Sidak's honestly significant difference test for multiple comparisons was used to determine significant differences among groups. The significant level used was *P* < 0.05.

## Author Contributions

A.T. and M. P. wrote the main manuscript. A.T. and N.P. carried out larvae rearing and sampling. M.G. and G.F. made cortisol measurements. P.K. carried out histology experiments and prepared figure 1. A.T. prepared figures 2–7. All authors reviewed the manuscript.

## Figures and Tables

**Table 1:** Morphological characteristics of European sea bass larvae collected at various stages during early ontogeny

Development	Description	DPH	Total Length (mm)
Embryos	70% of embryos in 50% epiboly stage	−2	
Hatching	70% of embryos are hatched	0	
Mouth opening	Mouth opens, complete yolk sac absorption	6	4.71 ± 0.09
First feeding	First day of exogenous feeding	9	5.25 ± 0.19
Flexion	65% completed the notochord flexion	29	11.1 ± 0.44
Fins	All fins have been developed	44	15.48 ± 0.21
